# ﻿Additions to *Hohenbuehelia* (Basidiomycota, Pleurotaceae): two new species and notes on *H.tristis* from northern Thailand

**DOI:** 10.3897/mycokeys.99.105317

**Published:** 2023-08-21

**Authors:** Monthien Phonemany, Santhiti Vadthanarat, Bhavesh Raghoonundon, Naritsada Thongklang, Olivier Raspé

**Affiliations:** 1 School of Science, Mae Fah Luang University, Chiang Rai 57100, Thailand; 2 Center of Excellence in Fungal Research, Mae Fah Luang University, 333 M.1 Thasud, Mueang, Chiang Rai 57100, Thailand; 3 Department of Biological Science, Faculty of Science, Ubon Ratchathani University, Ubon Ratchathani, Thailand

**Keywords:** Agaricales, DNA sequence heteromorphisms, molecular phylogeny, pleurotoid mushrooms, Southeast Asia, taxonomy, two new species

## Abstract

Two new species and a first geographical record of *Hohenbuehelia* are described from Thailand. Macroscopic and microscopic descriptions with photoplates, as well as a multigene phylogeny are provided. *Hohenbueheliaflabelliformis***sp. nov.** is recognised by large flabelliform basidiomata, densely villose yellowish-white pileus with white hairs near the point of attachment, basidiospores that mostly are ellipsoid in front view and phaseoliform in side view, the absence of cheilocystidia, and a trichoderm pileipellis. *Hohenbuehelialageniformis***sp. nov.** is characterised by fleshy basidiomata, velutinous pileus with whitish hairs near the point of attachment and the margin, elsewhere pale greyish-yellow and with only sparse white hairs, pale brown to light brown and mucilaginous context, subglobose basidiospores, lageniform cheilocystidia, an ixotrichoderm pileipellis, and the absence of pileoleptocystidia. *Hohenbueheliatristis* is characterised by small creamy-white, spathuliform basidiomata that are larger than the type subspecies, minutely pubescent pileus with tiny greyish hairs that disappear when mature, leaving the surface glutinous, faintly translucent and shiny, ellipsoid to sub-ellipsoid basidiospores, lecythiform to sublageniform cheilocystidia, and an ixotrichoderm pileipellis. *Hohenbueheliatristis* is recorded for Thailand for the first time. Based on the polymorphism observed in part of the nrLSU gene, the presence of two divergent lineages within *H.tristis* is discussed.

## ﻿Introduction

*Hohenbuehelia* Schulzer belongs to the family Pleurotaceae Kühner in the order Agaricales Underw. In former studies, the asexual stages of *Hohenbuehelia* species were separately placed in the genus *Nematoctonus* Drechsler (Drechsler 1941; Thorn and Barron 1986). Following the One Fungus = One Name nomenclatural rule, both the asexual and sexual stages were placed under *Hohenbuehelia* (Taylor 2011; McNeill et al. 2012; Thorn 2013), with *H.petaloides* (Bull.) Schulzer as the type species. Currently, 126 taxon names are listed under *Hohenbuehelia* in Index Fungorum (http://www.indexfungorum.org/), for 50 accepted species (He et al. 2019; Wijayawardene et al. 2022). The typical characteristics of this genus are pleurotoid, gelatinous basidiomata, white basidiospores with a germ pore, lecythiform cheilocystidia (if present) and thick-walled metuloid pleurocystidia (Stevenson 1964; Thorn and Barron 1986; Corner 1994; Silva-Filho and Cortez 2017; Holec and Zehnálek 2020). *Hohenbuehelia* and *Pleurotus* (Fr.) P. Kumm. have some similar morphological characters. However, *Hohenbuehelia* is distinguished by the synapomorphic gelatinous layer in the context under the pileipellis, which is absent in *Pleurotus* (Mentrida 2016). Most *Hohenbuehelia* species are decomposers and widely distributed in temperate and tropical areas (Læssøe and Petersen 2019). *Hohenbuehelia* species have been found growing on dead branches, decayed wood, logs, and sometimes on the bark of living trees or on herbaceous stems (Holec and Zehnálek 2020).

A few *Hohenbuehelia* species have been reported as edible. However, they have very low culinary value (Cruz and Gándara 2005). This genus is a good source of polyphenols and polysaccharides (Li et al. 2012; Li et al. 2017; Wang et al. 2018; Wang et al. 2019). Bioactive compounds extracted from some *Hohenbuehelia* species were found to have antioxidant properties as well as antitumor and antiviral activities (Ji et al. 2012; Sandargo et al. 2018a). Furthermore, the new derivatives thiopleurotinic acid A and thiopleurotinic acid B from extracts of *H.grisea* (Peck) Singer (strain MFLUCC 12-0451) were found to exhibit cytotoxicity towards a mouse fibroblast cell line, as well as antimicrobial activities (Sandargo et al. 2018a). Another compound, 4-hydroxypleurogrisein, was shown to inhibit hepatitis C virus infectivity in mammalian liver cells (Sandargo et al. 2018b). *Hohenbuehelia* sp. ZW-16 has been used for bioethanol production (Liang et al. 2013). Thorn et al. (2000) also found that the mycelia of the asexual stage of some *Hohenbuehelia* species are able to produce adhesive knobs that can capture nematodes. The diversity of bioactive compounds from *Hohenbuehelia* species and their potential applications underline the importance of detailed taxonomic study of this genus (Shipley et al. 2006; [Bibr B3][Bibr B40], 2018b).

Thailand has a high mushroom diversity with many new species yet to be discovered (Thongbai et al. 2018; Vadthanarat et al. 2021). Only four *Hohenbuehelia* species have been recorded from Thailand, namely *H.grisea*, *H.panelloides* Høiland, *H.petaloides* and *H.reniformis* (G. Mever & Fr.) Sing. (Chandrasrikul et al. 2011; Sandargo et al. 2018a). However, most of those reports did not provide detailed morphological descriptions nor molecular data in order to confirm the identifications (*H.grisea* was identified, based on ITS sequences only, without morphological data). In this study, during the survey of pleurotoid mushrooms in northern Thailand, several collections of *Hohenbuehelia* were obtained and studied. Based on morphological and phylogenetic results, two new species, *H.flabelliformis* and *H.lageniformis*, and the first record of *H.tristis* are described herein.

## ﻿Materials and methods

### ﻿Sample collection and morphological study

The mushroom specimens were collected during the rainy season in 2019 and 2020 from Chiang Mai and Chiang Rai Provinces, in northern Thailand. The fresh basidiomata were photographed in situ. Details including collecting date, locality, habitat and ecology of the surroundings, were noted. The specimens were wrapped in aluminium foil or kept in plastic boxes and brought back to the lab for morphological descriptions.

Macromorphological descriptions were done, based on the fresh specimens and colour codes were given following the colour charts of Kornerup and Wanscher (1978). The specimens were dried in a hot air dryer at 50 °C until the samples were completely dried and then kept separately in zip-lock plastic bags. The specimens were deposited in the Herbarium of Mae Fah Luang University (**MFLU**). Micromorphological characters were observed from the dried specimens. A razor blade was used to make thin sections of the specimens and these were mounted on slides in water, 5% potassium hydroxide (KOH) solution or 1% ammoniacal Congo red. The microcharacters were studied and photographed using a compound microscope Nikon Eclipse Ni. Freehand drawings were made for the microscopic features. Fifty basidiospores per basidioma were measured in side view. The notation [x/y/z] denotes the number of basidiospores (x) measured from the number of basidiomata (y) of the number of collections (z). At least 25 basidia, pleurocystidia, cheilocystidia, and pileipellis hyphae were observed and measured. The dimensions of microscopic structures are presented in the following format: (*a*–)*b*–*c*–*d* (−*e*), in which *c* represents the average, *b* the 5^th^ percentile, *d* the 95^th^ percentile, and minimum and maximum values *a* and *e* are shown in parentheses. *Q*, the length/width ratio, is presented in the same format. Facesoffungi numbers and MycoBank numbers are provided for each new species.

### ﻿DNA extraction and sequencing

Genomic DNA was extracted from the dried herbarium specimens using the Biospin Fungus Genomic DNA Extraction Kit (Bioer Technology, Hangzhou, China), following the manufacturer’s instructions. The ITS region and parts of the nrLSU and *tef*1 genes were amplified by a polymerase chain reaction (PCR) and sequenced. The following primers were used: ITS1-F and ITS4 for ITS (White et al. 1990; Gardes and Bruns 1993), LR0R and LR5 for nrLSU (Vilgalys and Hester 1990; White et al. 1990) and EF1-983F and EF1-1567R for *tef*1 (Rehner and Buckley 2005). The PCR cycling for ITS and LSU was as follows: 3 min at 94 °C; 35 cycles of 30 s at 94 °C, 30 s at 52 °C, 1 min at 72 °C; 10 min at 72 °C. For *tef*1, the following programme was used: 5 min at 95 °C; 35 cycles of 1 min at 94 °C, 2 min at 52 °C, 1.5 min at 72 °C; 10 min at 72 °C. The PCR-amplified products were purified and sequenced in forward and reverse directions, using PCR primers by Sangon Biological Engineering Technology & Services (Shanghai).

### ﻿Sequence alignment and phylogenetic analyses

Sequence reads were checked using Bioedit Sequence Alignment Editor version 7.0.9.0 and assembled using SeqMan (DNAstar, Madison, WI, USA). Each sequence was blasted using the Basic Local Alignment Search Tool (BLAST) against the National Center for Biotechnology Information (NCBI) database (http://www.ncbi.nlm.nih.gov/genbank/) to check that it was from the correct genus and not from contamination, as well as to find the closest matches. Newly-generated sequences were deposited in GenBank. All sequences (Table 1) including the outgroup were retrieved and aligned using MAFFT v.7 (Katoh et al. 2017) on the online server (http://mafft.cbrc.jp/alignment/server/). For *tef*1, introns were delimited by comparing with the amino acid sequence of a reference sequence and locating the GT/AG motifs of the splicing sites and removed for further analyses. The ITS and LSU alignments were trimmed separately using TrimAl to eliminate ambiguously aligned positions (Capella-Gutiérrez et al. 2009). The length of each character set was: ITS1+ITS2 = 445; LSU+5.8S = 995; TEF1 (exons) = 438. After checking for supported conflicts (BS ≥ 70%) between single-gene Maximum Likelihood (ML) phylogenies, a concatenated three-gene dataset was assembled.

**Table 1.** GenBank accession numbers and geographical origins of taxa used in the phylogenetic analyses.

Species	Specimen/culture	Country	GenBank accession numbers		
			ITS	nLSU	*tef*1
*Hohenbueheliaalgonquinensis*	RGT 870601/12 UWO (culture T-434)	Canada	KU355341	AF139950	KU355456
*H.angustata*	CBS 856.85	Canada	MH861919	MH873608	–
*H.atrocoerulea*	AMB 18080	Hungary	KU355304	KU355389	KU355439
*H.auriscalpium*	PRMJH1372007	Czech Republic	MT525862	MT534054	–
*H.bonii*	K(M):165700	England	KX064444	–	–
*H.boullardii*	JCM06005	Spain	MG553637	MG553644	–
*H.canadensis*, as H.atrocoeruleavar.grisea	DAOM 158848	Canada	KU355356	–	–
*H.carlothornii*	AMB 18106	Costa Rica	KY698012	KY698013	–
*H.chevallieri*	WU:6528	Austria	KT388040	–	–
*H.culmicola*	Roux 3488	Italy	KU355323	–	–
*H.cyphelliformis*	Z+ZT 994	Switzerland	KU355325	KU355393	KU355445
*H.faerberioides*	Mertens	France	MG553638	MG553645	MW240984
***H.flabelliformis***	**MFLU22-0008**	**Thailand**	**OP236779**	**OM521957**	**OM714821**
***H.flabelliformis***	**MFLU22-0009**	**Thailand**	**OP236780**	–	–
*H.fluxilis*	WU 29608	Austria	KU355326	–	–
*H.grisea*	MCVE 27293, as *H.myxotricha*	Italy	KU355329	KU355394	KU355447
*H.grisea*	VPI-F-0001921 (culture VT 1324 = T-132), as *H.nigra*	USA	KY679143	KY679143	–
*H.grisea*	MFLUCC 12-0451	Thailand	MF150036	–	–
*H.grisea*	HFJAU0029	China	MN258645	–	–
*H.ilerdensis*	Roux 3924	Spain	MG553639	MG553646	
*H.josserandii*	P.K. EG10-812-T-F	Germany	KU355353	KU355403	KU355463
***H.lageniformis***	**MFLU22-0010**	**Thailand**	**OP236781**	**OM521958**	**OM763737**
***H.lageniformis***	**MFLU22-0012**	**Thailand**	**OP236783**	**OM521959**	–
*H.leightonii*	WU 5846	Spain	MG553640	MG553647	–
*H.ligulata*	PDD:80775	New Zealand	KM975439	–	–
*H.longipes*	LIP 0400317	Italy	KU355333	KU355396	KU355449
*H.mastrucata*	TRTC 152314	Italy	KU355336	KU355397	KU355451
*H.mustialensis*	DAOM 46374	Canada	KY124252	–	–
*H.nimueae*	RGT 871128/01 UWO (culture T-489 = CBS 212.91), as *H.nigra*	Canada	KY679144	KY679144	–
*H.odorata*	TBGT17443	India	MN059651	–	–
*H.petaloides*	AMB 18088	Italy	KU355346	KU355402	KU355460
*H.pinacearum*	DAOM 84313	Canada	MH137814	MH137837	–
*H.portegna*	J.E. Wright 1136 BAFC	Argentina	AF139959	AF139959	–
***H.tristis***	**MFLU22-0015**	**Thailand**	**OP355451**	**OM521961**	–
***H.tristis***	**MFLU22-0016**	**Thailand**	**OP355450**	**OM521962**	**ON394004**
*H.reniformis*	HMJAU7091	China	GQ142024	GQ142041	–
*H.robusta*	CBS 130.68	–	MH859087	MH870800	–
*H.thornii*	WU 21790	Portugal	KU355343	KU355401	KU355457
*H.tremula*	M 0223665	Italy	KU355358	KU355406	KU355466
*H.tristis*	RV95/214 DUKE	Australia	–	AF042601	–
*H.tristis*	RV95/295 DUKE	Australia	–	AF135171	–
*H.unguicularis*	Z+ZT 1112	France	KU355361	KU355408	KU355467
*H.valesiaca*	Roux 2975	France	KU355340	KU355399	KU355455
*H.wilhelmii*	Z+ZT n. 1154	France	MF494947	MF494948	MF494949
*Pleurotuscitrinopileatus*	YAASM1585	China	KX836372	–	KX840311
*P.eryngii*	CCMSSC00480	China	KX836350	–	KX840226
*P.ostreatus*	TENN 53662 (= AFTOL-ID 564)	Austria	AY854077	AY645052	AY883432
*P.djamor*, as *P.placentodes*	HKAS51745	China	KR827693	KR827695	KR827699
*P.pulmonarius*	BCRC36906	Taiwan	MH453616	MH447275	MH500353

Notes: The newly-generated sequences in this study are presented in bold, “–” refers to the unavailability of sequence.

All phylogenetic analyses were done on the CIPRES Science Gateway version 3.3 web server (Miller et al. 2010), accessed at https://www.phylo.org/. For both Maximum Likelihood and Bayesian analyses, a mixed-model (partitioned) scheme was used, with the alignment divided in the following three character sets: ITS1+ITS2, LSU+5.8S, *tef*1. Maximum Likelihood phylogenetic inference was performed using RAxML-HPC2 version 8.2.12 (Stamatakis 2006) on XSEDE. Five *Pleurotus* species were used as outgroup. For Bayesian analysis, the best-fit substitution models were selected from jModelTest2 version 2.1.6 (Darriba et al. 2012) on XSEDE. The best-fit models were HKY+G for ITS1+ITS2, GTR+I+G for nrLSU+5.8S and SYM+G for *tef*1. Bayesian analysis was performed in MrBayes version 3.2.7a (Ronquist et al. 2012). Two runs of five chains each were run for 500,000 generations and sampled every 200 generations. The average standard deviation of split frequencies was 0.008720 at the end of the runs. The burn-in phase (25%) was estimated by checking the stationarity in the generation-likelihood plot in Tracer ver. 1.7.1 (Rambaut et al. 2018). The phylogenetic tree was visualised and edited in FigTree version 1.4.4 (Rambaut 2018).

## ﻿Results

### ﻿Phylogenetic analyses

**Figure 1.**
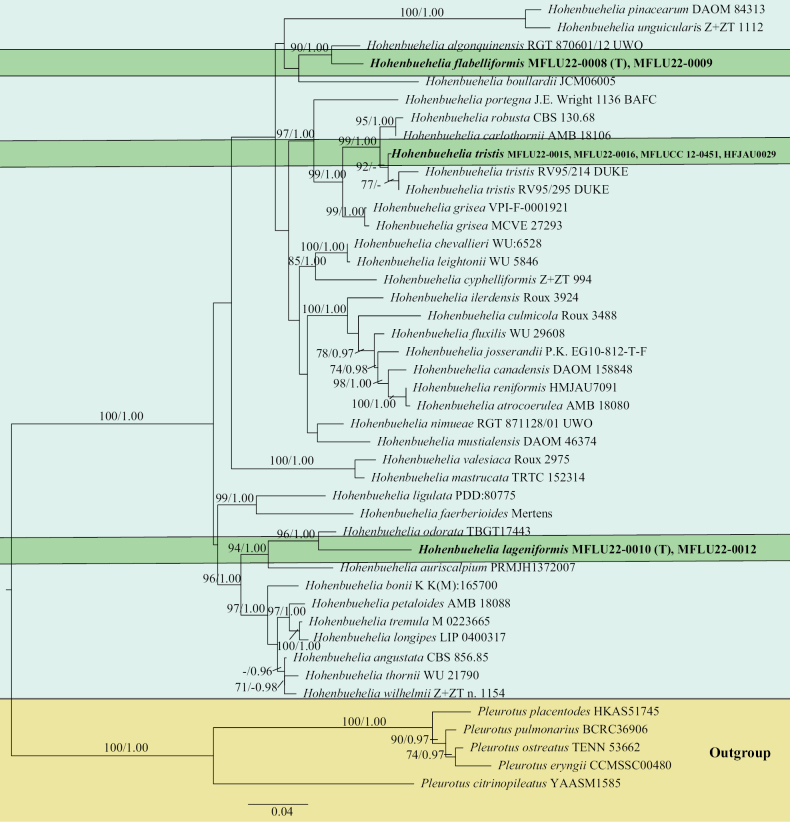
Phylogeny of selected sequences of *Hohenbuehelia* based on a Maximum Likelihood analysis of three nuclear gene regions (nrITS, nrLSU, *tef*1). The Maximum Likelihood bootstrap values (BS ≥ 70%) and Bayesian posterior probabilities values (PP ≥ 0.90) are shown on the branches. Newly-sequenced collections are in bold. Five *Pleurotus* species were used as outgroup. (T) designates holotypes. The sequence *H.flabelliformis* voucher number MFLU22-0008 was identical to MFLU22-0009 (ITS only). Sequences of *H.lageniformis* voucher number MFLU22-0010 were identical to MFLU22-0012 (ITS and LSU). Sequences of *H.tristis* voucher number MFLU22-0016 were identical to MFLU22-0015 (ITS and LSU) and to the two specimens MFLUCC 12-0451 and HFJAU0029, identified as *H.grisea* (both only ITS) from GenBank, except for three substitution heteromorphisms in the ITS sequence of MFLU22-0016 (see Table 2).

The combined dataset consisted of 39 *Hohenbuehelia* and five *Pleurotus* accessions (Table 1). The final alignment, including the gaps, was 1,878 characters long and was deposited in TreeBASE (submission ID 29653). The Bayesian and ML analyses resulted in similar tree topologies; thus, only the ML tree is shown with both Maximum Likelihood bootstrap (BS) values and Bayesian posterior probabilities (PP) (Fig. 1). In the phylogram, *H.flabelliformis* (MFLU22-0008 and MFLU22-0009) was closely related to *H.algonquinensis* (RGT 870601/12 UWO) from Canada with high support (90% BS, 1.00 PP). *Hohenbuehelialageniformis* (MFLU22-0010 and MFLU22-0012) was closely related to *H.odorata* (TBGT17443) from India with high statistical support (96% BS, 1.00 PP). The sequences of *H.tristis* (MFLU22-0015 and MFLU22-0016) are identical to two sequences from GenBank identified as *H.grisea* from Thailand (culture MFLUCC 12-0451) and China (HFJAU0029) with three substitution heteromorphisms and were closely related to *H.tristis* (RV95/214 DUKE and RV95/295 DUKE) from Australia with 92% BS, but low support in the BI analysis.

**Table 2.** Positions of substitution heteromorphisms in the ITS sequence of *H.tristis* MFLU22-0016 and corresponding character states in other *H.tristis* ITS sequences.

Specimen name/vouchers	Accession number	Position in alignment		
		147	552	588
*H.tristis* MFLU22-0015	OP355451	T	C	T
*H.tristis* MFLU22-0016	OP355450	K	Y	Y
*H.tristis* MFLUCC 12-0451(as *H.grisea* in GenBank)	MF150036	T	C	T
*H.tristis* HFJAU0029(as *H.grisea* in GenBank)	MN258645	T	C	T

### ﻿Taxonomy

#### ﻿ Hohenbuehelia flabelliformis

Taxon classificationFungiAgaricalesPleurotaceae

Phonemany & Raspé sp. nov.

98C53127-8E77-5CC6-8B74-F912A9EEA19D

MycoBank No: 843984

Facesoffungi Number: FoF10708

Figs 2 , 3

##### Diagnosis.

This species is distinguished from other *Hohenbuehelia* species by large flabelliform basidiomata, yellowish-white pileus that is densely villose with white hairs longer near the point of attachment, and shorter towards the margin, ellipsoid basidiospores, absence of cheilocystidia, and a trichoderm pileipellis.

**Figure 2.**
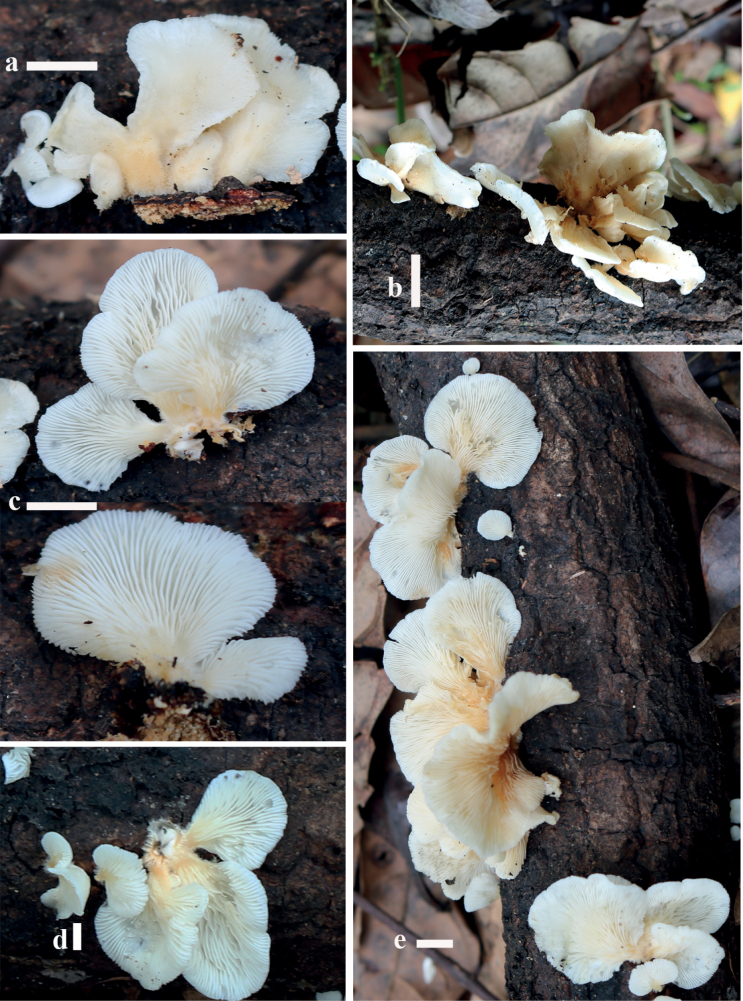
Basidiomata of *Hohenbueheliaflabelliformis* in the field **a, c–e** MFLU22-0008 **b** MFLU22-0009. Scale bars: 1 cm (**a–e**).

##### Holotype.

Thailand, Chiang Mai Province, Mae Taeng District, Pha Daeng Village, 27 May 2019, Monthien Phonemany (MFLU22-0008).

**Figure 3.**
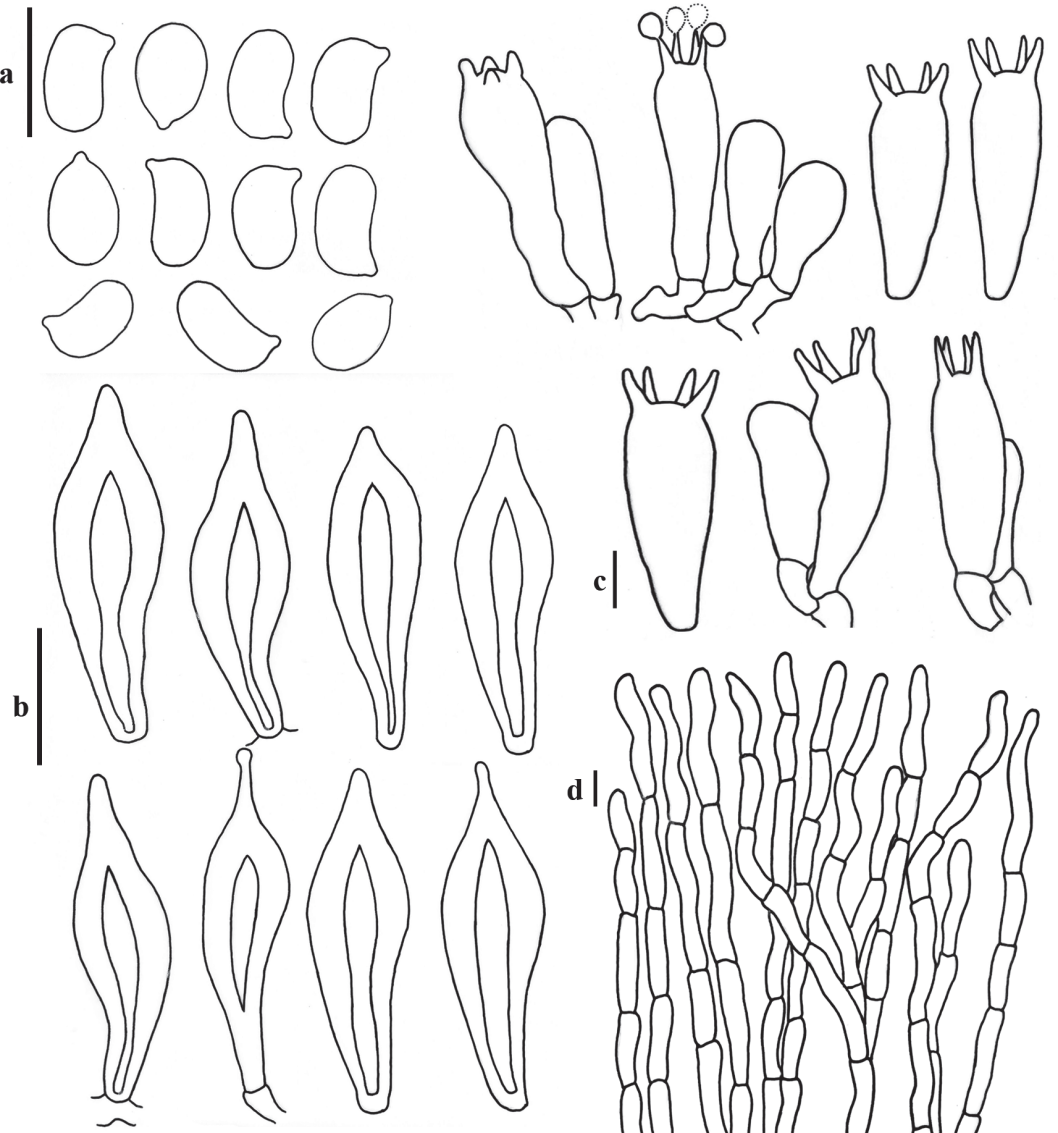
Micromorphology of *Hohenbueheliaflabelliformis* (MFLU22-0008, MFLU22-0009) **a** basidiospores **b** pleurocystidia **c** basidia and basidioles **d** pileipellis. Scale bars: 10 µm (**a, c**); 20 µm (**b, d**).

##### Etymology.

"*flabelliformis*" refers to the flabelliform shape of basidiomata.

##### Description.

***Pileus*** 35–45 × 20–40 mm, spathulate when young, expanding to spathuliform, flabelliform, rounded flabelliform or orbicular, white when young, becoming yellowish-white to pale yellow (4A2–4A3) at the centre and cream (2A3–2B3) to slightly darker elsewhere in age; surface densely villose with white hairs that are longer near the attachment and shorter towards the margin, as observed with a lens; margin white, incurved when young, becoming straight when old. ***Lamellae*** 1–3 mm wide, decurrent, pale yellow to yellowish-white (4A3–4A2), moderately crowded when mature, with lamellulae in 1–3 tiers; edge concolorous to sides, fimbriate. ***Stipe*** absent or as pseudostipe 5–12 mm × 3–8 mm. ***Context*** consisting of two layers: 1) non-gelatinous layer, 1 mm thick, soft when young and rather leathery when old, white to dirty white (4A1–4A2); 2) gelatinous layer 0.5 mm thick, soft, sticky, colourless. ***Odour*** mild, pleasant. ***Taste*** none. ***Spore print*** white.

***Basidiospores*** [150/3/2] (5.8–)6–7–8(–8.6) × (3.5–)4–4.2–5(–5.1) µm, *Q* = (1.3–)1.36–1.67–2.01(–2.03), ellipsoid to elongate (oblong) in side view, smooth, thin-walled, inamyloid. ***Basidia*** (21–)21–25.8–35(–37) × (5–)5.3–7.3–10.5(–11) µm, subclavate to clavate, with 4 sterigmata 4–8 µm long, hyaline, smooth, thin-walled. ***Cheilocystidia*** absent. ***Pleurocystidia*** metuloidal, present on both sides of lamellae and visible with a lens, (34–)34–42–54(–55) × (8.5–)8.5–11–14(–14.5) µm, scattered, narrowly fusiform to fusiform, mucronate at apex, brownish in KOH. ***Hymenophoral trama*** irregular, hyphae 2–5 µm wide. ***Pileipellis*** a trichoderm, hyaline in KOH, brownish in water, with cylindrical terminal elements 34–86 × 4–7 µm. ***Pileoleptocystidia*** absent. ***Pileus trama*** consists of two different layers: 1) upper layer gelatinous, composed of horizontally arranged, smooth, colourless encrusted hyphae, 2–5 µm wide; 2) lower layer, non-gelatinous, composed of interwoven, smooth, hyaline hyphae, 2–4 µm wide. ***Clamp connections*** present in pileipellis, pileus trama, and hymenophoral trama.

##### Habitat and distribution.

On dead wood, scattered or fasciculate by 2–4 basidiomata. So far only found in tropical forests of northern Thailand.

##### Additional specimens examined.

Thailand. Chiang Rai Province, Pa Daed District, Pa Ngae Village, 9 August 2019, Monthien Phonemany (MFLU22-0009).

##### Notes.

The basidiomata colour of *Hohenbueheliaflabelliformis* is similar to *H.angustata* (Berk.) Singer, *H.bonii* A.M. Ainsw., *H.concentrica* Corner, *H.carlothornii* Consiglio, Setti & Thorn, *H.horrida* (Boedijn) Corner, *H.luteola* G. Stev, *H.malesiana* Corner, *H.odorata* C.K. Pradeep & Bijeesh, *H.olivacea* Yu Liu & T. Bau, and *H.testudo* (Berk.) Pegler. The basidiomata range from white, yellowish-white, yellow-brown, to pinkish-orange, and are spathulate to flabelliform. *Hohenbueheliaangustata*, originally described from Brazil, differs from *H.flabelliformis* by its smaller, smooth, greyish-yellow basidiomata, with stipe 4.5 mm long, smaller basidiospores (3.5–5 × 2.5–3.5 µm), smaller cheilocystidia, and the presence of pileocystidia (Silva-Filho and Cortez 2017). *H.horrida* and *H.odorata* differ from *H.flabelliformis* by smaller basidiospores (5.2–7.6 × 4.8–6.4 µm), lack of cheilocystidia, and presence of pileoleptocystidia (Bijeesh et al. 2019). *Hohenbueheliatestudo* differs by smaller basidia (20–25 × 5–6 µm), larger pleurocystidia (44–78 × 12–18 µm) with thick yellowish walls, and the presence of cheilocystidia (Corner 1994). *Hohenbueheliamalesiana*, described from Brazil, is different from *H.flabelliformis* by having longer, subcylindrical basidiospores (7–9 × 3.5–4 µm), pileipellis as an interrupted cutis, and presence of cheilocystidia (Corner 1994). *Hohenbueheliabonii*, from England, has larger yellow-brown basidiomata (20–75 mm diam.), smooth pileus surface, larger basidiospores (7.2–10.4 × 4.5–6.1 µm), larger pleurocystidia (56–103 × 11–19 µm), and an ixotrichoderm or ixocutis pileipellis (Ainsworth et al. 2016). *Hohenbueheliacarlothornii* described from Costa Rica, is different by having off-white basidiomata with a large pseudostipe (20–32 × 14–25 mm), presence of cheilocystidia (Consiglio et al. 2018b). *Hohenbueheliaconcentrica* from Singapore, has larger basidiomata (80 mm wide), larger basidiospores (8–8.5 × 6–6.7 µm), and absence of cheilocystidia (Corner 1994). *Hohenbueheliaincarnata*, from the Solomon Islands, differs from *H.flabelliformis* by subglobose basidiospores and the presence of subcylindrical to submoniliform cheilocystidia (Corner 1994). *Hohenbueheliaolivacea*, originally described from China, has reniform basidiomata with dense and long tomentum, light brown to pallid brown in gelatinous zone, and the presence of cheilocystidia (Liu and Bau 2009).

Phylogenetically, *H.flabelliformis* was closely related to *H.algonquinensis* Consiglio, Setti & Thorn. (voucher RGT 870601/12 UWO) with 3.5% (21/599) differences in the ITS sequence, 1.4% (12/839) in the LSU sequence, and 6.66% (35/540) in the *tef*1 sequence. Moreover, the morphologies of both species are completely different, with *H.algonquinensis* having glossy black pileus, ungulate to dimidiate basidiomata and contrasting white or off-white lamellae (Consiglio et al. 2018b).

#### ﻿ Hohenbuehelia lageniformis

Taxon classificationFungiAgaricalesPleurotaceae

Phonemany & Raspé sp. nov.

B27429FD-2A21-5A2C-A8CE-62C1441E7BA5

MycoBank No: 843985

Facesoffungi Number: FoF10709

Figs 4 , 5

##### Diagnosis.

This species is distinguished from other *Hohenbuehelia* species by having velutinous pileus with whitish hairs near the point of attachment, and at the margin, elsewhere pale greyish-yellow, and with only sparse white hairs, pale brown to light brown and mucilaginous context, subglobose to ellipsoid basidiospores, lageniform cheilocystidia, an ixotrichoderm pileipellis, and the absence of pileoleptocystidia.

**Figure 4.**
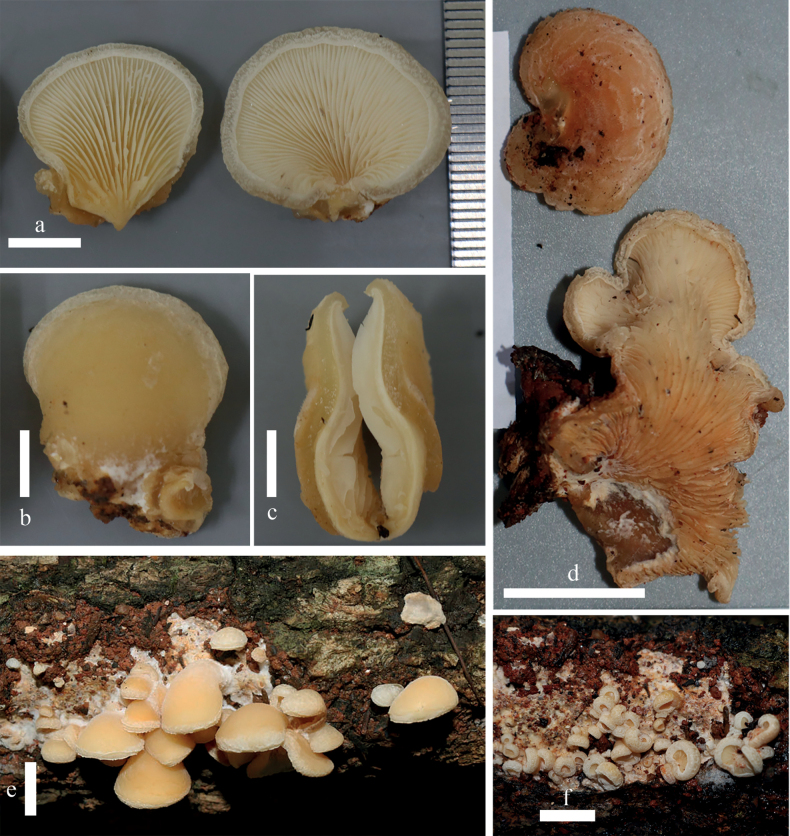
Basidiomata of *Hohenbuehelialageniformis* on the substrate and detailed photographs in the lab **a–c** MFLU22-0010 **d** MFLU22-0012 **e, f** MFLU22-0014. Scale bars: 1 cm (**a–e**); 2 cm (**f**).

##### Holotype.

Thailand, Chiang Mai Province, Mae On District, Huai Kaew Subdistrict, Pok Village, 29 June 2019, Monthien Phonemany (MFLU22-0010).

**Figure 5.**
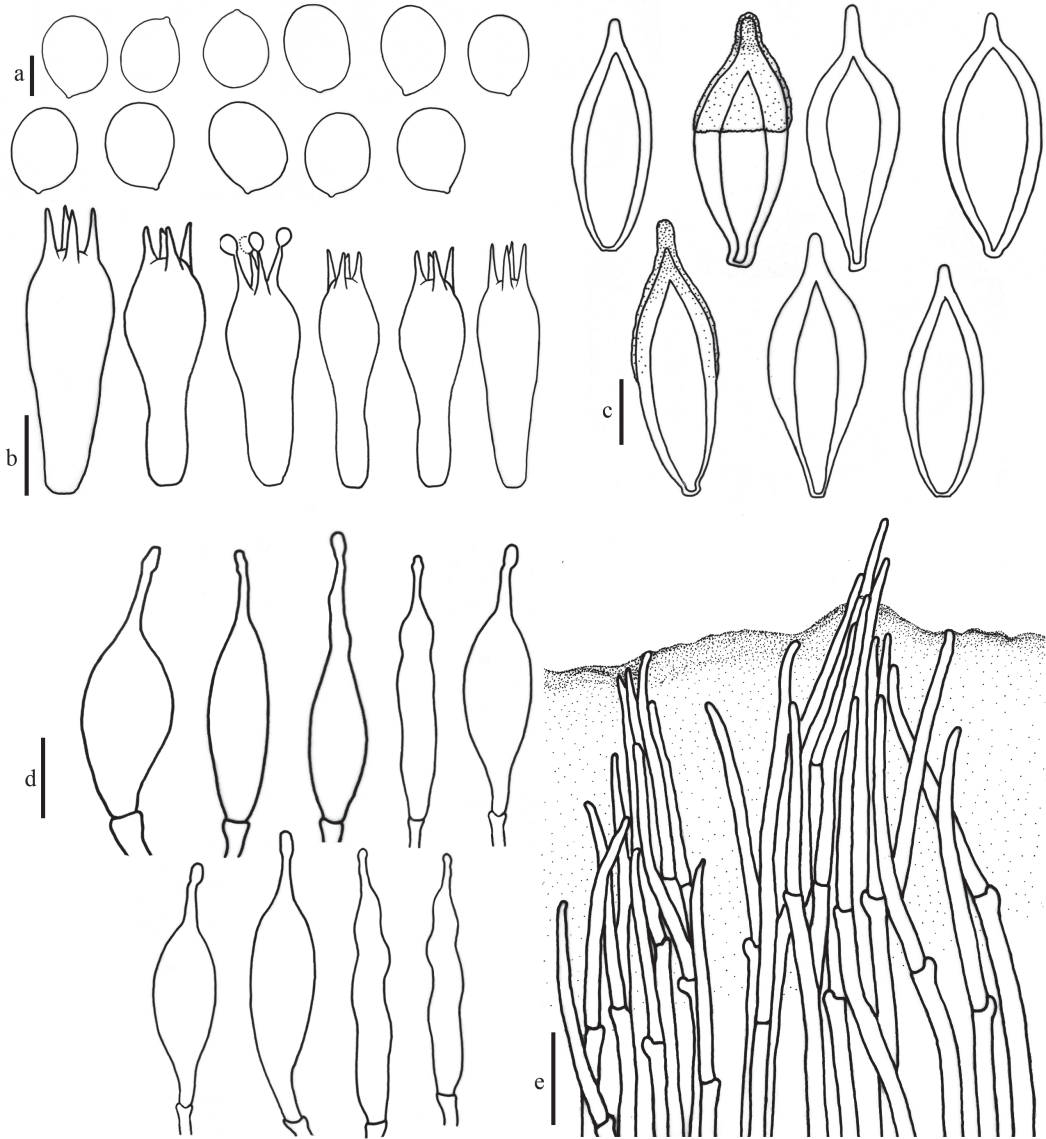
Micromorphology of *Hohenbuehelialageniformis* (MFLU22-0010, MFLU22-0012, MFLU22-00104) **a** basidiospores **b** basidia **c** pleurocystidia **d** cheilocystidia **e** pileipellis. Scale bars: 5 µm (**a**); 20 µm (**b, c**); 10 µm (**d**); 50 µm (**e**).

##### Etymology.

"*lageniformis*" refers to the lageniform shape of cheilocystidia.

##### Description.

***Pileus*** 25–30 × 15–25 mm, spathuliform when young, rounded flabelliform to sub-rounded flabelliform when mature, light yellow (4A4), pale orange to orange white (5A2–5A3), greyish-orange (5B4) becoming darker with age, sometimes sessile or with laterally attached stipe; surface moist, shiny, velutinous with whitish (1A1) hairs near the point of attachment, elsewhere light yellow to pale greyish-yellow (4A4–4B4); margin discolorous from pileus, velutinous with whitish hairs as observed under the lens, incurved even when mature, entire or sometimes undulate when old. ***Lamellae*** 1–2 mm wide, decurrent, white to pale orange (5A1–5A2) becoming slightly dark in age, close to moderately crowded, in 2–3 tiers. ***Stipe*** absent or sometimes with pseudostipe 0.5–1 mm long when young and disappearing when mature. ***Context*** consisting of two layers: 1) non-gelatinous layer, 1 mm thick, fleshy, white (1A1); 2) gelatinous layer, 1–2 mm thick, soft, sticky, brownish-orange to light brown (5C5–5D5). ***Odour*** mild. ***Taste*** none. ***Spore print*** white.

***Basidiospores*** [150/3/3] (6.9–)7–8.8–10(–10.5) × (5.5–)6–7.0–8(–8.3) µm, *Q* = (0.99–)1.11–1.26–1.49(–1.53) subglobose to ellipsoid in front view, ellipsoid to phaseoliform in side view, smooth, thin-walled, inamyloid. ***Basidia*** (24.3–)24–31.9–53(–58.2) × (5.4–)5.5–9.4–14(–14.6) µm, clavate, with (2)–4 sterigmata, 4–8 µm long, hyaline, smooth, thin-walled. ***Cheilocystidia*** (17.6–)18–21.9–26.5(–26.9) × (3.2–)3–5.0–9(–8.9) µm, lageniform with an inflated base with a thin, rostrate apex, hyaline, thin-walled. ***Pleurocystidia*** (31–)31–50.3–70(–71) × (9.7–)10–15.3–20(–20.3) µm, metuloidal, setiform, narrowly fusiform to fusiform, encrusted with crystals, brownish or yellowish in water, colourless, but still encrusted with crystals in KOH. ***Hymenophoral trama*** subregular, hyphae 2–4 µm wide. ***Pileipellis*** an intricate trichoderm with cylindrical terminal elements 41–122 × 2–5 µm. ***Pileoleptocystidia*** absent. ***Pileus trama*** consisting of two layers: 1) upper layer gelatinous, composed horizontally arranged, smooth, colourless, encrusted hyphae, 1–4 µm wide; 2) non-gelatinous layer, composed interwoven, smooth with hyaline hyphae, 2–5 µm wide. ***Clamp connections*** present in pileipellis, pileus trama and hymenophoral trama.

##### Habitat and distribution.

Solitary, gregarious to imbricate, on decaying branches in a tropical forests in northern Thailand.

##### Additional specimens examined.

Thailand. Chiang Rai Province, Muang Chiang Rai District, Mae Yao Subdistrict, Huai Mae Sai Village, 10 July 2019, Monthien Phonemany (MFLU22-0011); ibid., 10 July 2019, Monthien Phonemany (MFLU22-0012); ibid., 10 July 2019, Monthien Phonemany (MFLU22-0013); Pa Daed District, Pa Ngae Village, 7 September 2019, Monthien Phonemany (MFLU22-0014).

##### Notes.

*Hohenbuehelialageniformis* is characterised by velutinous pileus with whitish hairs near the point of attachment and at the margin, elsewhere pale greyish-yellow and with only sparse white hairs, subglobose to ellipsoid basidiospores, and the absence of pileoleptocystidia. *Hohenbueheliaangustata*, *H.bonii*, *H.carlothornii*, *H.concentrica*, *H.flabelliformis*, *H.horrida*, *H.luteola*, *H.mellea* Corner, and *H.odorata* have similar pileus colour, for example, pale orange to orange white, light yellow, yellowish-white, yellow-brown, honey-yellow or ochraceous brownish. However, those species have significant morphological differences. *Hohenbueheliaangustata* differs from *H.lageniformis* by having a smooth pileus surface, serrate margin, smaller basidiospores (3.5–5 × 2.5–3.5 µm), cutis pileipellis (Silva-Filho and Cortez 2017). *Hohenbueheliabonii* differs by having a smooth pileus surface and larger pleurocystidia (56–103 × 11–19 µm) (Ainsworth et al. 2016). *Hohenbueheliacarlothornii* differs by having finely white-tomentose pileus, smaller basidiospores (7–8.2 × 3.1–3.7 µm), smaller basidia (19–24 × 5.2–6.8 µm), smaller cheilocystidia (13–17 × 4.9–9.4 µm), and larger pleurocystidia (55–63 × 14–18 µm) (Consiglio et al. 2018b). *Hohenbueheliaconcentrica* has larger basidiomata (80 mm wide), ovoid basidiospores (8–8.5 × 6–6.7 µm), and no cheilocystidia (Corner 1994). *Hohenbueheliahorrida* has pale grey to greyish-brown lamellae, larger basidiomata (70 mm wide), smaller basidiospores (5–6 × 3–4 µm), smaller basidia (18–25 × 3–4 µm), and larger pleurocystidia (140 × 12.5 µm) (Corner 1994). *Hohenbuehelialuteola* differs by smaller basidiospores (8–9 × 4.5–5 μm), and smaller pleurocystidia (45–50 ×15–20 µm) (Stevenson 1964). *Hohenbueheliamellea* has smaller basidiospores (5–6.5 × 2–3.5 µm), larger cheilocystidia (30–50 × 8–18 µm), and larger pleurocystidia (50–160 × 12–20 µm) (Corner 1994). *Hohenbueheliaflabelliformis* has thinner gelatinous layer (0.5 mm), smaller basidiospores (6–8 × 4–5 µm), smaller pleurocystidia (34–54 × 8.5–14 µm), and no cheilocystidia. *Hohenbueheliaodorata* differs by having smaller basidiospores (5.2–7.6 × 4.8–6.4 µm), smaller cheilocystidia (15–23.5 × 3–7 µm), smaller pleurocystidia (28.5–49 × 10–14.5 µm), and presence of cylindrical to flexuous pileoleptocystidia (Bijeesh et al. 2019).

In the phylogenic tree, the most closely-related species to *H.lageniformis* was *H.odorata* (voucher TBGT17443). However, the genetic distance between the ITS sequence of *H.lageniformis* and *H.odorata* was 4.62% (27/584), which supports the distinction of the two species. Moreover, these two species also show morphological differences (see above).

#### ﻿ Hohenbuehelia tristis

Taxon classificationFungiAgaricalesPleurotaceae

G. Stev.

73D7AFED-3038-5FD2-AE0E-17EF3A25B5CB

MycoBank No: 332010

Figs 6 , 7

##### Remarks.

The following description is based solely on the Thai materials we collected and examined.

##### Description.

***Pileus*** 15–20 × 20–30 mm, spathuliform to reniform, dimidiate to orbicular, greyish-white (1B1) to yellowish-white (1B2) when young, becoming white (1A1) in age, glutinous, very faintly translucent, shiny when mature; upper surface minutely pubescent with greyish hairs (5A1–5B1) near the point of attachment and more sparsely so towards the margin as observed with a lens, with hairs disappearing in age; margin incurved becoming upturned in age. ***Lamellae*** radiating from point of attachment, 1 mm broad, very crowded, white (1A1) to pale yellow (1A3); lamellulae in 1–4 tiers. ***Stipe*** absent or pseudostipe sometimes present, laterally or dorsally attached, 1 mm long when young, then disappearing when old. ***Context*** consisting of two layers: 1) leathery layer, 1 mm thick; 2) gelatinous layer, 1–2 mm thick, soft, sticky, colourless. ***Odour*** and ***taste*** not observed. ***Spore print*** white.

**Figure 6.**
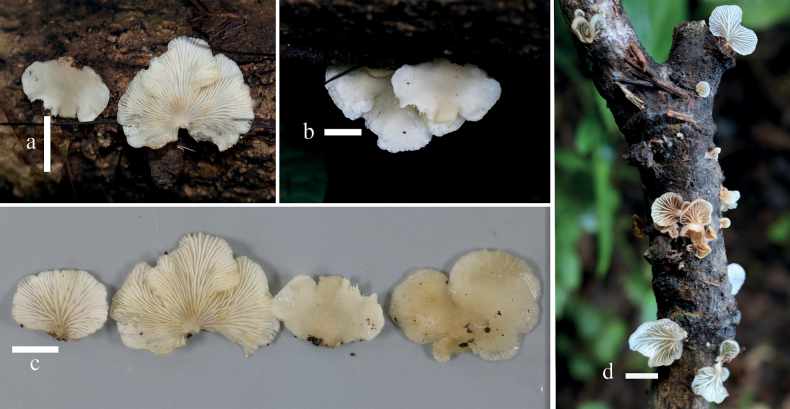
Basidiomata of *Hohenbueheliatristis* in the field **a–c** MFLU22-0015 **d** MFLU22-0016. Scale bars: 1 cm (**a–d**).

***Basidiospores*** [150/3/2] (5.1–)6–6.8–8(–9) × (3.5–)3.5–4.0–5(–5.2), *Q* = (1.21–)1.38–1.70–2.08(–2.26), ellipsoid, sub-ellipsoid to elongate, smooth, thin-walled, inamyloid. ***Basidia*** (13–)14–21.2–24(–24.2) × (5–)5.5–6.6–7(–7) µm, clavate to subcylindrical, mostly with four sometimes with two sterigmata, 2–4 µm long, hyaline, smooth, thin-walled. ***Cheilocystidia*** (10.9–)11–13.1–19(–19.8) × (4.2–)4.4–5.5–6.5(–6.5) µm, lecythiform to sublageniform, with subcapitate to capitate apex, hyaline, thin-walled. ***Pleurocystidia*** (38–)38–61.5–82(–82) × (10.7–)11–15.0–18.5(–18.6) µm, subfusiform, narrowly fusiform to fusiform, brownish, encrusted with crystals when observed in water, but crystals disappearing in KOH. ***Hymenophoral trama*** subregular, hyphae 2–4 µm wide. ***Pileipellis*** as tufts of ixotrichoderm with cylindrical terminal elements 40–105 × 3–5.5 µm, with light brown intracellular pigments. ***Pileoleptocystidia*** absent. ***Pileus trama*** consisting of two layers: 1) upper layer gelatinous, composed of horizontally arranged, smooth, colourless, encrusted hyphae, 1.5–2.5 µm wide; 2) lower layer non-gelatinous, composed of interwoven, smooth and hyaline hyphae, width 2–4 µm. ***Clamp connections*** present in pileipellis, pileus trama and hymenophoral trama.

**Figure 7.**
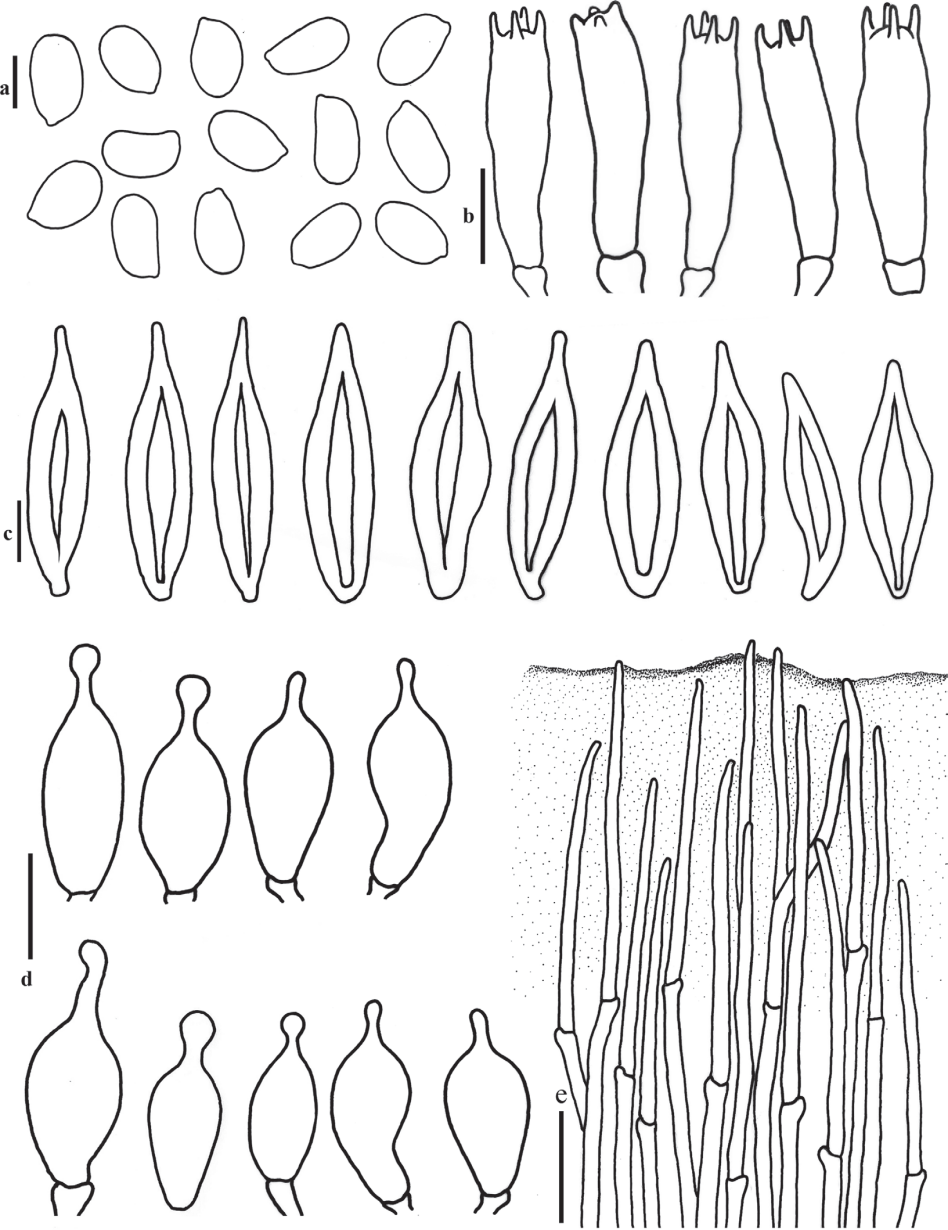
Micromorphology of *Hohenbueheliatristis***a** basidiospores **b** basidia **c** pleurocystidia **d** cheilocystidia **e** pileipellis (MFLU22-0015, MFLU22-0016). Scale bars: 5 µm (**a**), 10 µm (**b, d**), 20 µm (**c**), 50 µm (**e**).

##### Habitat.

Solitary, gregarious to imbricate, on dead small branches.

##### Specimens examined.

Thailand. Chiang Rai Province, Muang Chiang Rai District, Nang Lae Nai Village, 31 July 2019, Monthien Phonemany (MFLU22-0015). Chiang Rai Province, Muang Chiang Rai District, Mae Yao subdistrict, Huai Mae Sai Village, 31 July 2019, Monthien Phonemany (MFLU22-0016).

##### Notes.

Two accessions identified as *H.grisea*, the culture MFLUCC 12-0451 from Thailand and HFJAU0029 from China (unpublished), had the same ITS sequence than *H.tristis* (MFLU22-0015 and MFLU22-0016) except for three substitution heteromorphisms in the ITS sequence of MFLU22-0016 (see Table 2). The two former sequences retrieved from GenBank have no corresponding morphological descriptions available as evidence. Therefore, these might have been wrongly identified, since ITS sequences are identical to sequences obtained from our collections of *H.tristis* (except for the heteromorphisms detailed in Table 2). Additional confirmation of the taxonomic identity of our specimens was obtained by comparing the morphology of our specimens with *H.grisea* which was originally described as Pleurotusatrocoeruleusvar.griseus Peck from New York. The latter is distinguished by a greyish to greyish-brown, sparsely tomentose pileus, the lamellae becoming cream-coloured in age (Peck 1891). *Hohenbueheliatristis* is characterised by reniform basidiomata, minutely pubescent pileus with greyish hairs that disappear when mature, leaving the surface gelatinous, faintly translucent and shiny, ellipsoid to sub-ellipsoid basidiospores, lecythiform to sublageniform cheilocystidia, and an ixotrichoderm pileipellis. *Hohenbueheliatristis* described from New Zealand differs from our collections (MFLU2022-0015 and MFLU2022-0016) by having smaller basidiomata (10–20 × 10–15 mm), smaller (mostly narrower) basidiospores (7 × 3 µm), larger and pseudo-amyloid metuloids (80–90 × 15–20 µm), pileipellis as tufts of parallel larger hyphae (3–8 µm in diam.) (Stevenson 1964). In our phylogenetic analysis (Fig. 1), the Thai accessions of *H.tristis* formed a monophyletic group with the accessions of *H.tristis* from New Zealand. The morphological differences we observed between the Thai and New Zealand accessions suggested that they might not be conspecific. Additionally, the single synapomorphic position we observed in the LSU sequence (position 685 in the alignment; see Table 3) is not incompatible with two distinct species, since a genetic distance of only one substitution can be observed between other closely-related species (e.g. *H.tremula* and *H.longipes*; Table 4). However, the LSU sequence of the Thai specimen MFLU22-0016 showed two to three heteromorphisms corresponding to the differences between the other Thai specimens and the New Zealand specimens (Table 3). This suggests either incomplete lineage sorting or that the two lineages can still interbreed. Heteromorphisms were also observed in the ITS sequence of MFLU22-0016, but we could not compare with ITS sequences of materials from New Zealand, which were unavailable. In view of all the heteromorphisms, we decided not to treat the Thai materials as a new species distinct from *H.tristis*.

**Table 3.** Position of substitutions and substitution heteromorphisms in the LSU sequence of *H.tristis* MFLU22-0015, MFLU22-0016, and corresponding character states in *H.tristis* RV95/214 DUKE, RV95/295 DUKE.

Specimen name/vouchers	Accession number	Position in the alignment			
		658	663	664	666
*H.tristis* MFLU22-0015	OP355451	C	G	T	G
*H.tristis* MFLU22-0016	OP355450	Y	G	Y	R
*H.tristis* RV95/214 DUKE	AF042601	T	A	T	A
*H.tristis* RV95/295 DUKE	AF135171	T	G	C	A

**Table 4.** Genetic distance (number of substitutions, excluding heteromorphisms) between LSU sequences of closely-related *Hohenbuehelia* species.

Species name/ specimen number	1	2	3	4	5	6	7	8	9
1. *H.tristis* MFLU22-0015									
2. *H.tristis* MFLU22-0016	0								
3. *H.atrocoerulea* AMB 18080	13	13							
4. *H.carlothornii* AMB 18106	3	3	14						
5. *H.reniformis* HMJAU7091	11	11	2	12					
6. *H.robusta* CBS 130.68	3	3	14	0	12				
7. *H.tristis* RV95/214 DUKE	3	2	15	5	13	5			
8. *H.tristis* RV95/295 DUKE	3	1	14	4	12	4	2		
9. *H.longipes* LIP 0400317	24	24	15	23	13	23	24	23	
10. *H.tremula* M 0223665	25	25	16	24	14	24	25	24	1

## ﻿Discussion

The Pleurotaceae belong to the Agaricales and comprise the monophyletic pleurotoid genera *Pleurotus* and *Hohenbuehelia* (Thorn et al. 2000; Koziak et al. 2007). *Hohenbuehelia* species have often been misidentified, in part because holotypes are missing or because types of species put in synonymy were not adequately studied (Consiglio et al. 2018b). In the past, most *Hohenbuehelia* species were introduced, based only on short morphological descriptions (e.g., Peck 1891; Coker 1944; Stevenson 1964). Consiglio (2016, 2017a, b) and Consiglio and Setti (2017) designated lectotypes, neotypes, and epitypes to clarify older species names or names that lack modern and molecularly-characterised holotypes. For example, the holotype of *H.tristis* was identified by Stevenson (1964) without any molecular evidence. Later, an nrLSU sequence of *H.tristis* was obtained for the first time by Moncalvo et al. (2000). The heteromorphisms we observed in the nrLSU and ITS sequences of one of the Thai specimens related to *H.tristis* suggested that interbreeding may occur between two divergent lineages within *H.tristis*. Although more data would be needed to confirm it, we hypothesise that those two lineages diverged in geographical isolation (between Southeast Asia and Oceania) and then came in contact in Southeast Asia. This kind of biogeographical history, revealed by DNA sequence variation, have been observed in other Agaricales, for example, *Agaricussubrufescens* Peck (Chen et al. 2016).

Some of the recently-described species were still introduced, based on only single-gene molecular evidence. In this study, we provide multiple-gene sequence data and detailed descriptions supporting the introduction of two new *Hohenbuehelia* species and a note on *H.tristis* from Thailand. At present, a total of six *Hohenbuehelia* species have been reported from Thailand including the ones in this study and three that were previously reported, namely *H.panelloides*, *H.petaloides*, and *H.reniformis* (Chandrasrikul et al. 2011). The report of *H.grisea* by Sandargo et al. (2018a) has to be excluded since we showed that the correct identification of their material is *H.tristis*. More studies on *Hohenbuehelia* are needed to clarify their taxonomy and more new species might be discovered.

## Supplementary Material

XML Treatment for Hohenbuehelia flabelliformis

XML Treatment for Hohenbuehelia lageniformis

XML Treatment for Hohenbuehelia tristis

## ﻿Additional information

### Author ORCIDs

Monthien Phonemany https://orcid.org/0000-0002-7865-4659

Santhiti Vadthanarat https://orcid.org/0000-0002-9035-0375

Bhavesh Raghoonundon https://orcid.org/0000-0001-6671-2404

Naritsada Thongklang https://orcid.org/0000-0001-9337-5001

Olivier Raspé https://orcid.org/0000-0002-8426-2133

### Data availability

All of the data that support the findings of this study are available in the main text, or in publicly accessible data repositories, as indicated in the text.

## References

[B1] Ainsworth AM Suz LM Dentinger BTM (2016) *Hohenbueheliabonii* sp. nov. and *H.culmicola*: Two pearls within the Marram Oyster. Field Mycology: a Magazine for the Study and Identification of Wild Fungi 17(3): 78–86. 10.1016/j.fldmyc.2016.07.004

[B2] Bijeesh C Manoj Kumar A Pradeep CK Vrinda KB (2019) A new species of *Hohenbuehelia* (Pleurotaceae) from India. Phytotaxa 420: 056–064. 10.11646/phytotaxa.420.1.3

[B3] Bohni N Schumpp O Schnee S Bertrand S Gindro K Wolfender JL (2013) Targeted isolation of induced and bioactive metabolites from fungal co-cultures. Planta Medica 79(13). 10.1055/s-0033-1351867

[B4] Capella-Gutiérrez S Silla-Martínez JM Gabaldón T (2009) trimAl: A tool for automated alignment trimming in large-scale phylogenetic analyses. Bioinformatics 25(15): 1972–1973. 10.1093/bioinformatics/btp348 19505945 PMC2712344

[B5] Chandrasrikul A Suwanarit P Sangwanit U Lumyong S Payapanon A Sanoamuang N Pukahuta C Petcharat V Sardsud U Duengkae K Klinhom U (2011) Checklist of mushrooms (Basidiomycetes) in Thailand. Office of Natural Resources and Environmental Policy and Planning, Bangkok, Thailand, 448 pp.

[B6] Chen J Moinard M Xu J Wang S Foulongne-Oriol M Zhao R Hyde KD Callac P (2016) Genetic analyses of the internal transcribed spacer sequences suggest introgression and duplication in the medicinal mushroom *Agaricussubrufescens*. PLoS ONE 11(5): e0156250. 10.1371/journal.pone.0156250 PMC4882077 27228131

[B7] Coker WC (1944) The smaller species of *Pleurotus* in North Carolina. Elisha Mitchell Scientific Society 60: 71–95.

[B8] Consiglio G (2016) Nomenclatural novelties: *Agaricus*, *Hohenbuehelia*, *Pleurotus* typifications. Index Fungorum : Published Numbers 292: 1–2.

[B9] Consiglio G (2017a) Nomenclatural novelties: *Agaricuscyphelliformis* epitype, *A.niger* neotype, hic designatus; *Hohenbueheliapseudopetaloides*, *H.thornii* spp. nov.; *Pleurotusauriscalpium*, *P.longipes* lectotypes, hic designatus. Index Fungorum: Published Numbers 326: 1.

[B10] Consiglio G (2017b) Nomenclatural novelties: *Agaricusvalesiacus* epitype, *Pleurotusauriscalpium* epitype, *P.longipes* epitype, hic designatus. Index Fungorum: Published Numbers 327: 1.

[B11] Consiglio G Setti L (2018a) The Genera *Hohenbuehelia* and *Resupinatus* in Europe / I Generi *Hohenbuehelia* e *Resupinatus* in Europe. Monografie di Pagine di Micologia (Vol. 3). Associazione Micologica Bresadola, Trento, 448 pp.

[B12] Consiglio G Setti L (2017) Nomenclatural novelties. Index Fungorum: Published Numbers 325: 1.

[B13] Consiglio G Setti L Thorn RG (2018b) New species of *Hohenbuehelia*, with comments on the *Hohenbueheliaatrocoerulea* – *Nematoctonusrobustus* species complex. Persoonia 41(1): 202–212. 10.3767/persoonia.2018.41.10 30728605 PMC6344808

[B14] Corner EJH (1994) On the Agaric genera *Hohenbuehelia* and *Oudemansiella* Part I: *Hohenbuehelia*. Gardens’ Bulletin (Singapore) 46: 1–47.

[B15] Cruz V Gándara E (2005) El género *Hohenbuehelia* (Basidiomycotina, Agaricales, Tricholomataceae) en Veracruz, México. Revista Mexicana de Micología 21: 29–37.

[B16] Darriba D Taboada GL Doallo R Posada D (2012) jModelTest 2: More models, new heuristics and parallel computing. Nature Methods 9(8): e772. 10.1038/nmeth.2109 PMC4594756 22847109

[B17] Drechsler C (1941) Some hyphomycetes parasitic on free-living terricokms nematodes. Phytopathology 31: 773–802.

[B18] Gardes M Bruns TD (1993) ITS primers with enhanced specificity for basidiomycetes ‐ application to the identification of mycorrhizae and rusts. Molecular Ecology 2(2): 113–118. 10.1111/j.1365-294X.1993.tb00005.x 8180733

[B19] He MQ Zhao RL Hyde KD Begerow D Kemler M Yurkov A McKenzie EHC Raspé O Kakishima M Sánchez-Ramírez S Vellinga EC Halling R Papp V Zmitrovich IV Buyck B Ertz D Wijayawardene NN Cui BK Schoutteten N Liu XZ Li TH Yao YJ Zhu XY Liu AQ Li GJ Zhang MZ Ling ZL Cao B Antonín V Boekhout T Da Silva BDB De Crop E Decock C Dima B Dutta AK Fell JW Geml J Ghobad-Nejhad M Giachini AJ Gibertoni TB Gorjón SP Haelewaters D He SH Hodkinson BP Horak E Hoshino T Justo A Lim YW Menolli Jr N Mešić A Moncalvo JM Mueller GM Nagy LG Nilsson RH Noordeloos M Nuytinck J Orihara T Ratchadawan C Rajchenberg M Silva-Filho AGS Sulzbacher MA Tkalčec Z Valenzuela R Verbeken A Vizzini A Wartchow F Wei T-Z Weiß M Zhao C-L Kirk PM (2019) Notes, outline and divergence times of Basidiomycota. Fungal Diversity 99(1): 105–367. 10.1007/s13225-019-00435-4

[B20] Holec J Zehnálek P (2020) Taxonomy of *Hohenbueheliaauriscalpium*, *H.abietina*, *H.josserandii*, and one record of *H.tremula*. Czech Mycology 72(2): 199–220. 10.33585/cmy.72204

[B21] Ji H Zhang L Zhang H Zhang G Wang Q Zhang H (2012) Antioxidant properties of ethanol extracts from *Hohenbueheliaserotina* and *Armillariamellea*. Applied Mechanics and Materials 195–196: 342–346. 10.4028/www.scientific.net/AMM.195-196.342

[B22] Katoh K Rozewicki J Yamada K (2017) MAFFT online service: Multiple sequence alignment, interactive sequence choice and visualization. Briefings in Bioinformatics 30: 772–780. 10.1093/bib/bbx108 PMC6781576 28968734

[B23] Kornerup A Wanscher JH (1978) Methuen Handbook of Colour (3^rd^ edn.). Eyre Methuen, London.

[B24] Koziak ATE Kei CC Thorn RG (2007) Phylogenetic analyses of Nematoctonus and *Hohenbuehelia* (Pleurotaceae). Canadian Journal of Botany 85(8): 762–773. 10.1139/B07-083

[B25] Læssøe T Petersen JH (2019) Fungi of Temperate Europe (Vol. 1). Princeton University Press, Princeton and Oxford.

[B26] Li X Wang Z Wang L Walid E Zhang H (2012) Ultrasonic-assisted extraction of polysaccharides from *Hohenbueheliaserotina* by response surface methodology. International Journal of Biological Macromolecules 51(4): 523–530. 10.1016/j.ijbiomac.2012.06.006 22728641

[B27] Li X Wang L Wang Z (2017) Structural characterization and antioxidant activity of polysaccharide from *Hohenbueheliaserotina*. International Journal of Biological Macromolecules 98: 59–66. 10.1016/j.ijbiomac.2016.12.089 28069348

[B28] Liang X Hua D Wang Z Zhang J Zhao Y Xu H Li Y Gao M Zhang X (2013) Production of bioethanol using lignocellulosic hydrolysate by the white rot fungus *Hohenbuehelia* sp. ZW-16. Annals of Microbiology 63(2): 719–723. 10.1007/s13213-012-0524-6

[B29] Liu Y Bau T (2009) A new species of *Hohenbuehelia* from China. Mycotaxon 108(1): 445–448. 10.5248/108.445

[B30] Liu Y Bau T Li TH (2010) Three new records of the genus *Hohenbuehelia* in China. Mycosystem 29: 454–458.

[B31] McNeill J Barrie FR Buck WR Demoulin V Greuter W Hawksworths DL Herendeen PS Knapp S Marhold K Prado J Van Reine WFP Smith GF Wiersma JH Turland NJ (2012) International Code of Nomenclature for algae, fungi and plants (Melbourne Code) adopted by the Eighteenth International Botanical Congress Melbourne, Australia, July 2011. Regnum Vegetabile 154.

[B32] Mentrida CS (2016) Species delimitation and phylogenetic analyses of the genus *Hohenbuehelia* in central Europe. (Doctoral dissertation, UniWien).

[B33] Miller A Chen J Takasuka TE Jacobi JL Kaufman PD Irudayaraj JMK Kirchmaier AL (2010) Proliferating Cell Nuclear Antigen (PCNA) is required for cell cycle-regulated silent chromatin on replicated and nonreplicated genes. The Journal of Biological Chemistry 285(45): 35142–35154. 10.1074/jbc.M110.166918 20813847 PMC2966128

[B34] Moncalvo JM Lutzoni FM Rehner SA Johnson J Vilgalys R (2000) Phylogenetic relationships of agaric fungi based on nuclear large subunit ribosomal DNA sequences. Systematic Biology 49(2): 278–305. 10.1093/sysbio/49.2.278 12118409

[B35] Peck CH (1891) Annual report of the State Botanist. Annual Report of the Trustees of the State Museum of Natural History 44: 117–187.

[B36] Rambaut A (2018) FigTree v. 1.4.4. http://tree.bio.ed.ac.uk/software/figtree/

[B37] Rambaut A Drummond AJ Xie D Baele G Suchard MA (2018) Posterior summarisation in Bayesian phylogenetics using Tracer 1.7. Systematic Biology 67(5): 901–904. 10.1093/sysbio/syy032 29718447 PMC6101584

[B38] Rehner SA Buckley E (2005) A Beauveria phylogeny inferred from nuclear ITS and EF1-α sequences: Evidence for cryptic diversification and links to *Cordyceps* teleomorphs. Mycologia 97(1): 84–98. 10.3852/mycologia.97.1.84 16389960

[B39] Ronquist F Teslenko M Van Der Mark P Ayres DL Darling A Höhna S Larget B Liu L Suchard MA Huelsenbeck JP (2012) Mrbayes 3.2: Efficient bayesian phylogenetic inference and model choice across a large model space. Systematic Biology 61(3): 539–542. 10.1093/sysbio/sys029 22357727 PMC3329765

[B40] Sandargo B Thongbai B Stadler M Surup F (2018a) Cysteine-Derived Pleurotin Congeners from the Nematode-Trapping Basidiomycete *Hohenbueheliagrisea*. Journal of Natural Products 81(2): 286–291. 10.1021/acs.jnatprod.7b00713 29356520

[B41] Sandargo B Thongbai B Praditya D Steinmann E Stadler M Surup F (2018b) Antiviral 4-hydroxypleurogrisein and antimicrobial pleurotin derivatives from cultures of the nematophagous basidiomycete *Hohenbueheliagrisea*. Molecules 23(10): e2697. 10.3390/molecules23102697 PMC6222660 30347707

[B42] Shipley SM Barr AL Graf SJ Collins RP McCloud TG Newman DJ (2006) Development of a process for the production of the anticancer lead compound pleurotin by fermentation of *Hohenbueheliaatrocoerulea*. Journal of Industrial Microbiology & Biotechnology 33(6): 463–468. 10.1007/s10295-006-0089-0 16501932

[B43] Silva-Filho AGS Cortez VG (2017) *Hohenbuehelia* (Pleurotaceae) in western Paraná, Brazil. Acta Biológica Paranaense 46: 23–38. 10.5380/abpr.v46i0.54587

[B44] Singer R (1951) New Genera of Fungi. VII. Lilloa 22: e255. 10.1080/00275514.1951.12024157

[B45] Stamatakis A (2006) RAxML-VI-HPC: Maximum likelihood-based phylogenetic analyses with thousands of taxa and mixed models. Bioinformatics 22(21): 2688–2690. 10.1093/bioinformatics/btl446 16928733

[B46] Stevenson G (1964) The Agaricales of New Zealand: V. Kew Bulletin 19(1): 1–59. 10.2307/4108283

[B47] Taylor JW (2011) One Fungus = One Name: DNA and fungal nomenclature twenty years after PCR. IMA Fungus 2(2): 113–120. 10.5598/imafungus.2011.02.02.01 22679595 PMC3359808

[B48] Thongbai B Hyde KD Lumyong S Raspé O (2018) High undescribed diversity of Amanita section Vaginatae in northern Thailand. Mycosphere: Journal of Fungal Biology 9(3): 462–494. 10.5943/mycosphere/9/3/3

[B49] Thorn RG (2013) Nomenclatural novelties. Index Fungorum: Published Numbers 16: 1.

[B50] Thorn RG Barron GL (1986) *Nematoctonus* and the tribe Resupinateae in Ontario, Canada. Mycotaxon 25: 321–453.

[B51] Thorn RG Moncalvo JM Reddy CA Vilgalys R (2000) Phylogenetic analyses and the distribution of nematophagy support a monophyletic Pleurotaceae within the polyphyletic pleurotoid-lentinoid fungi. Mycologia 92(2): 241–252. 10.1080/00275514.2000.12061151

[B52] Vadthanarat S Halling RE Amalfi M Lumyong S Raspé O (2021) An unexpectedly high number of new *Sutorius* (Boletaceae) species from Northern and Northeastern Thailand. Frontiers in Microbiology 12: e643505. 10.3389/fmicb.2021.643505 PMC8072293 33912149

[B53] Vilgalys R Hester M (1990) Rapid genetic identification and mapping of enzymatically amplified ribosomal DNA from several *Cryptococcus* species. Journal of Bacteriology 172(8): 54238–54246. 10.1128/jb.172.8.4238-4246.1990 PMC213247 2376561

[B54] Wang L Li X Wang B (2018) Synthesis, characterization and antioxidant activity of selenium modified polysaccharides from *Hohenbueheliaserotina*. International Journal of Biological Macromolecules 120: 1362–1368. 10.1016/j.ijbiomac.2018.09.139 30261249

[B55] Wang L Li X Wang B (2019) The cytotoxicity activity of *Hohenbueheliaserotina* polyphenols on HeLa cells via induction of cell apoptosis and cell cycle arrest. Food and Chemical Toxicology 124: 239–248. 10.1016/j.fct.2018.12.001 30529121

[B56] White TJ Bruns T Lee STJ (1990) Amplification and direct sequencing of fungal ribosomal RNA genes for phylogenetics. In: Innis MA Gelfand DH Sninsky JJ White TJ (Eds) PCR protocols: a guide to methods and applications.Academic Press, San Diego, 315–322. 10.1016/B978-0-12-372180-8.50042-1

[B57] Wijayawardene NN Hyde KD Dai DQ Sánchez-García M Goto BT Saxena RK Erdoğdu M Selçuk F Rajeshkumar KC Aptroot A Błaszkowski J Boonyuen N da Silva GA de Souza FA Dong W Ertz D Haelewaters D Jones EBG Karunarathna SC Kirk PM Kukwa M Kumla J Leontyev DV Lumbsch HT Maharachchikumbura SSN Marguno F Martínez-Rodríguez P Mešić A Monteiro JS Oehl F Pawłowska J Pem D Pfliegler WP Phillips AJL Pošta A He MQ Li JX Raza M Sruthi OP Suetrong S Suwannarach N Tedersoo L Thiyagaraja V Tibpromma S Tkalčec Z Tokarev YS Wanasinghe DN Wijesundara DSA Wimalaseana SDMK Madrid H Zhang GQ Gao Y Sánchez-Castro I Tang LZ Stadler M Yurkov A Thines M (2022) Outline of Fungi and fungus-like taxa – 2021. Mycosphere: Journal of Fungal Biology 13(1): 53–453. 10.5943/mycosphere/13/1/2

